# Primary Care Physicians’ Personal and Professional Attributes Associated With Forgoing Own Care and Presenteeism: A Cross Sectional Study

**DOI:** 10.3389/ijph.2021.1604442

**Published:** 2022-02-15

**Authors:** Christine Cohidon, Liv Mahler, Barbara Broers, Thierry Favrod-Coune, Amir Moussa, Paul Sebo

**Affiliations:** ^1^ Family Medicine Department, Center for Primary Care and Public Health (Unisanté), University of Lausanne, Lausanne, Switzerland; ^2^ Primary Care Division, Geneva University Hospitals, Geneve, Switzerland; ^3^ Primary Care Unit, University of Geneva, Geneva, Switzerland

**Keywords:** primary care physician, illness behaviour, forgoing care, forgoing sick leave, presenteeism

## Abstract

**Objective:** The aim of this study was to describe the prevalence of forgoing care and forgoing sick leave among primary care physicians (PCPs) in Switzerland and to investigate associated factors.

**Methods:** A random sample of 1,000 PCPs in French-speaking regions of Switzerland (participation rate: 50%) was asked whether they had forgone care and sick leave during the last year. Sociodemographic, personal and occupational characteristics were recorded. Logistic regressions were performed to study these behaviours.

**Results:** 37% of respondents reported at least one episode of forgoing care and 29% reported an episode of forgoing sick leave. No associations were found between individual characteristics and forgoing care. A heavy workload was the most common reason evoked for forgoing care. Coming to work when sick (presenteeism) was associated with female sex, younger age, having a chronic illness, working in a suburban area and working full-time.

**Conclusion:** A high proportion of PCPs in Switzerland is forgoing own care and continues to work despite sickness. New generations of PCPs should require careful monitoring, and specific solutions should be sought to reduce these harmful behaviours.

## Introduction

According to the “Quintuple aim” defined to guide healthcare organizations in directing their efforts, one part of the healthcare system’s mission is ensuring the well-being of its workforce [1, 2]. Indeed, sick healthcare professionals negatively affect healthcare systems through lower staff retention, enhanced recruitment needs, lower productivity and efficiency, and ultimately, poorer quality of care and patient safety [3]. Yet, healthcare professionals, especially physicians, seem to be particularly negligent of their own health [4–6]. This behaviour has been described before [4, 7, 8] and seems to be shared by physicians in the public and private sectors [9]. The literature clearly shows that general practitioners (GPs) tend not to consider themselves as patients [10–12].

How physicians neglect their health can be illustrated via several specific behaviours, known as illness behaviours, including forgoing care (defined as not seeking healthcare while presenting medical symptoms) [7, 13], self-treatment [14, 15], a reluctance to take sick leave [3, 7] and not having their own GP [13]. Forgoing care has mainly been studied in the context of the psychological distress linked to physicians’ substantial exposure to occupational stress [16], particularly among GPs [17, 18]. Yet, physicians’ difficulties accepting and dealing with their somatic ailments seem to be compounded when their ailments are psychological, and this could have significant consequences on the quality of care they provide to others [19]. Physical health should not be forgotten either [6]. Regarding self-treatment, Montgomery’s literature review reported self-medication rates of over 50% in three-quarters of the 27 studies analysed [14]. More recently, in a country neighbouring Switzerland, Schulz’s study reported a self-medication rate of 60% among German GPs [20]. Finally, not only can physicians be reluctant to seek care, they also seem to be hesitant to take sick leave [4, 7, 9]. The prevalence of this behaviour, often termed presenteeism, may be 80–90% among physicians in comparison with 30–70% among other professions [9]. Finally, according to the literature, only about 50% of physicians have their own GP [14], and there are large variations in this rate [13]. Schulz reported that only 19% of German GPs had their own doctor [20], whereas rates in the general population in Western countries are generally over 75% in some countries.

GPs’ conscious and unconscious opinions and attitudes might help to explain these illness behaviours [21]. GPs may feel uncomfortable in the role of the patient and fear that other colleagues will interpret their need for help as an indicator of their inability to cope [3, 22, 23]. Other reported explanations include workload and lack of time, stigma [19], confidentiality, the fear of being seen as incompetent (by peers or patients) [10, 17, 19], embarrassment about the triviality of their condition and cultural myths about physicians’ invincibility [6, 13, 16, 17, 19, 24]. Finally, financial reasons have also been given by physicians working in private practice [13].

In a context where many Western countries are reporting a shortage of GPs [25], this issue may be particularly critical. Kay’s literature review underlined the paucity and poor quality of data about this topic [13]. Schulz et al. mentioned the need to analyse specific reasons for GPs’ illness behaviour [20]. Studies on this topic often treat physicians as a homogenous group, paying little attention to potential differences in their sociodemographic/personal or professional characteristics: existing reviews in this field rarely distinguish between different medical specialities [13, 14, 24]. Preventing GPs’ harmful illness behaviours and proposing interventions for change require identifying factors associated with them.

The present study aimed to describe the prevalence of forgoing care and forgoing sick leave in primary care physicians (PCPs) in Switzerland and to investigate sociodemographic, personal health and professional characteristics associated with these illness behaviours.

## Methods

### Study Design and Population

The present study was based on a large, cross-sectional survey on the health of PCPs (general practitioners, paediatricians and gynaecologists) in French-speaking regions of Switzerland (representing about one third of Switzerland’s 8.5 million inhabitants) in autumn 2020. A sample of 1,000 PCPs was randomly drawn from the French-speaking part of the Swiss Medical Association list (N = 2,455 PCPs practicing in this area). This list contains about 95% of the PCPs in Switzerland. A postal letter invited the selected PCPs to complete the survey via an internet link to an electronic questionnaire. They could also complete a paper version of the paper questionnaire and return it by post. A reminder was sent to non-responders 1 month after the first letter. No incentives were offered for participation. The Regional Research Ethics Committee (CCER) approved this study (Reference 2019-01850).

### Data

The questionnaire included several parts:

- Sociodemographic characteristics i.e., sex, age group, marital status and nationality;
- Personal health characteristics: perceived health [excellent, very good, good, moderate or poor-for the analyses we included it as continuous variable], having one’s own GP [yes, no], having seen one’s PCP or a psychiatrist/psychologist in the past 12 months [yes, no], medical history of main chronic physical and psychological diseases over the five last years and current treatments (see Supplementary Appendix). Regarding illness, we created a three level variable on the existence of a chronic illness, distinguishing between psychological and physical illnesses [yes physical illness/yes psychological illness/no].
- Practice and professional characteristics: medical speciality [general internal medicine, paediatrics, gynaecology], practice size [solo, duo, group practice, other], rural/urban area, number of years worked in private practice, number of half-days worked per week and participation in on-call duty (at the hospital or at the practice) [yes, no]. Urbanity level was defined based on the practice postal code, and then categorized (three levels) according to the typology of municipalities (communes) established by the Swiss Federal Statistical Office (FSO).
- Forgoing care was investigated using the following question: “In the last 12 months, did you ever plan to visit a general practitioner or specialist for a health problem (or a check-up) and then eventually decide not to do so?” [yes, no]. In cases where care had been forgone in the last year, the following answers were available (multiple choices possible): “I was too busy,” “I treated the problem by myself,” “I did not need a medical consultation in the end,” “Other” (to be specified). Forgoing sick leave was investigated using the following question: “In the last 12 months, did you ever consider taking a few days (or more) off work for health reasons and then eventually give up?” [yes, no]. In cases where sick leave had been forgone in the last year, the following answers were available (multiple choices possible): “I did not want to abandon my patients,” “I wanted to avoid financial loss,” “I did not need sick leave in the end,” “Other” (to be specified).

### Statistical Analysis

First, we carried out descriptive statistics (frequencies). We compared the proportions of forgoing care, forgoing sick leave and reasons for forgoing care and sick leave in subgroups of PCPs, according to gender, age and speciality, using chi-squared tests.

Second, associations between forgoing care or forgoing sick leave (as dependent variables) and PCPs’ sociodemographic, personal health and professional characteristics (as independent variables) were estimated using logistic regression models. In a first step, associations with PCPs’ sociodemographic, personal health and professional characteristics were assessed using univariate logistic regression models. Variables associated with outcomes (with *p*-values ≤ 0.2) were then included in a multivariate model. Backward stepwise selection was used to obtain the final model that contained all variables with *p* values ≤ 0.05, as well as gender and age.

Although we found no associations between forgoing care and any of the independent variables through univariate logistic regressions, we did observe significant differences according to why physicians forwent care; thus, we modelled forgoing care’s reasons to study their associations with the independent variables. We created a new variable opposing forgoing care due to a work overload *vs.* forgoing care for other reasons.

The number of missing data was very small and therefore we did not use the usual techniques recommended for handling missing data, such as mean substitution or imputation. We simply omitted these cases and analyzed the remaining data. All statistical analyses were performed using Stata Software (StataCorp LLC).

## Results

The characteristics of the 503 PCPs (50% participation rate) who completed the questionnaire are presented in Table 1: 51% were female, 72% were <60 years old (y.o.), two-thirds were GPs and one-third were paediatricians or gynaecologists.

**TABLE 1:** Study population characteristics (N = 503) – [the Swiss Physicians’ Health Study, French-speaking Switzerland 2021].

Personal characteristics	N	% or median (IQR)
Sex		
Female	258	51.3
Male	245	48.7
Age group		
<45 years old	139	27.6
45–54 years old	147	29.2
55–64 years old	142	28.2
>64 years old	75	14.9
Marital status		
Single	69	13.8
Married	352	70.2
Divorced or separated	74	14.8
Widowed	6	1.2
Nationality		
Swiss	317	63.1
Naturalised Swiss citizen	117	23.3
Other	68	13.5
Existing chronic illness	248	49.4
Perceived health		
Excellent	90	18.0
Very good	192	38.5
Good	187	37.5
Moderate or poor	30	6.0
Have their own doctor	237	47.4
Practice & professional characteristics		
Medical speciality		
General internal medicine	333	66.9
Paediatrics	95	19.1
Gynaecology	70	14.0
Type of practice		
Solo	171	34.1
Duo	105	20.9
Group	226	45.0
Location of practice		
Urban	210	41.7
Suburban	191	38.0
Rural	102	20.3
Number of years working in private practice	487	13 (16)
Number of half-days worked per week	500	8 (2)
Participation in on-call duty	248	49.6

At least one episode of forgoing care in the previous year was reported by 37% of PCPs, with no differences regarding sex, age or medical speciality, and 69% of them stated that this was because of a heavy workload. About half of PCPs reported that they had self-managed their illness (29%) or that a medical consultation had not been necessary in the end (21%). Demographic differences were observed in the reasons why care was forgone: women and younger PCPs were significantly more likely than men and older PCPs, respectively, to report that forgoing care was due to a work overload (79% women *vs.* 57% men, *p* < 0.01; and 75% PCPs <45 y.o. *vs.* 23% PCPs >64 y.o, *p* < 0.01 global test) (Table 2).

**TABLE 2:** Causes of forgoing care and sick leave according to sex, age and speciality (frequencies) – [the Swiss Physicians’ Health Study, French-speaking Switzerland 2021].

	N^a^	All (%)	Sex (%)		Age (yrs, %)						Speciality^b^ (%)			
	500		Male	Female	*p* ^c^	<45	45–54	55–64	>64	*p* ^c^	GIM	Ped	Gyn	*p* ^c^
Forgoing care^d^	183	36.7	35.1	38.1	0.49	38.9	39.0	38.3	24.3	0.13	37.4	30.8	42.4	0.30
I was too busy	122	68.9	57.3	78.9	<0.01	75.5	73.7	72.0	23.5	<0.01	67.2	75.0	70.4	0.71
I treated the problem by myself	51	28.8	28.0	29.5	0.83	28.3	19.3	38.0	35.3	0.18	28.7	25.0	33.3	0.79
I did not need a medical consultation in the end	38	21.5	19.5	23.2	0.56	26.4	12.3	18.0	47.1	0.01	18.0	25.0	33.3	0.19
Forgoing sick leave^e^	145	29.1	24.8	33.1	0.04	34.5	27.4	32.6	14.9	0.02	30.6	25.5	27.3	0.60
I did not want to abandon my patients	97	66.9	55.0	75.3	0.01	79.2	50.0	69.6	63.4	0.03	68.9	62.5	61.1	0.71
I wanted to avoid financial loss	77	53.1	50.0	55.3	0.53	54.1	52.5	56.5	36.4	0.69	52.4	62.5	44.4	0.49
I did not need sick leave in the end	78	53.8	50.0	56.5	0.44	56.2	50.0	54.3	54.5	0.95	48.5	75.0	55.6	0.06

a Total number of respondents.

b GIM: general internal medicine, Ped: paediatrics, Gyn: gynecology.

c Chi-squared test.

d Other reasons for forgoing care, N = 15.

e Other reasons for forgoing sick leave, N = 18.

Forgoing sick leave in the last year was reported by 29% of PCPs, with a significantly higher frequency among women (33% *vs.* 25% in men, *p* = 0.04) and younger doctors (34% PCPs <45 y.o. *vs.* 15% PCPs >64 y.o., *p* = 0.02). The most cited reason for forgoing sick leave was that physicians did not want to abandon their patients (67%). This reason was more likely to be reported by women (75%) than men (55%, *p* = 0.01) and by physicians <45 y.o. (79% *vs.* 50% PCPs 45–54 y.o., *p* = 0.03 global test). Financial reasons were cited by 53% of the PCPs, with no differences regarding sex, age or medical speciality. Finally, 54% of the PCPs thought that they were able to work on regardless (Table 2).

Multivariate analyses showed no associations between forgoing care and personal or professional characteristics. Thus, we also modelled the reasons for forgoing care by opposing a heavy workload (reported at least once) and all other reasons, to study their predictive factors. Forgoing care because of a heavy workload was more frequent among women (OR = 2.04; 95% CI [0.97–4.29]), among physicians working in suburbs (OR = 2.47 [1.07–5.71]) and those with their own doctor (OR = 2.47 [1.18–5.15]), and it was lower among older PCPs (OR = 0.15 [0.04–0.64] in PCPs ≥65 y.o.) (Table 3). Results were quite similar regarding forgoing sick leave. According to our multivariate analyses, this behaviour was more frequent among women (OR = 1.71 [1.04–2.81]), physicians who had been naturalised as Swiss citizens (OR = 1.86 [1.10–3.12]) and physicians with chronic diseases (OR = 2.21 [1.38–3.53]), and it increased as physicians perceived their health to be poorer (OR = 2.73 [2.01–3.71]). The probability of forgoing sick leave also increased with the number of half-days worked weekly (OR = 1.14 [1.02–1.27]). In contrast, forgoing sick leave was less frequent among older PCPs (OR = 0.41 [0.17–0.95] in PCP ≥65 y.o.) (Table 4).

**TABLE 3:** Personal and professional factors associated with PCPs forgoing care due to heavy workloads (*vs.* forgoing care for other reasons, N = 183, logistic regression) – [the Swiss Physicians’ Health Study, French-speaking Switzerland 2021].

Characteristics	N	%^b^	Univariate analyses	Multivariate analyses Final model^c^ (N = 176)	
			OR [95% CI]	OR [95% CI]	*p*-value
Sex – Ref. Male	82	57.2	1	1	
Female	95	78.9	2.79 [1.44–5.39]	2.04 [0.97–4.29]	0.06
Age – Ref. <45 years old	53	75.5	1	1	
45–54	57	73.7	0.91 [0.38–2.15]	1.03 [0.42–2.58]	0.94
55–64	50	72.0	0.84 [0.35–2.01]	1.22 [0.47–3.16]	0.67
≥65 years old	17	23.5	0.10 [0.03–0.36]	0.15 [0.04–0.64]	0.36
Marital status – Ref. married	132	65.9	1		
Single	20	75.0	1.55 [0.53–4.54]		
Divorced	24	79.2	1.96 [0.69–5.61]		
Nationality – Ref. Swiss	108	64.8	1		
Naturalised Swiss citizen	45	73.3	1.49 [0.69–3.22]		
Other	24	79.2	2.06 [0.71–15.96]		
Perceived health – (Excellent to poor)^a^	176	—	1.41 [0.93–2.13]		
Existing chronic disease – Ref. No	70	72.9	1		
Yes, physical illness	51	56.9	0.49 [0.23–1.05]		
Yes, psychological illness	56	75.0	1.12 [0.50–2.49]		
Have their own doctor – Ref. No	93	60.2	1	**1**	
Yes	83	79.5	2.56 [1.31–5.04]	2.47 [1.18–5.15]	0.02
Setting – Ref. urban	66	60.6	1	1	
Suburb area	72	79.2	2.47 [1.16–5.25]	2.47 [1.07–5.71]	0.03
Rural area	39	64.1	1.16 [0.51–2.63]	1.07 [0.40–2.41]	0.97
Speciality – Ref. General internal medicine	122	67.2	1		
Paediatrics	28	75.0	1.46 [0.57–3.73]		
Gynaecology	27	70.4	1.16 [0.47–2.87]		
Practice type – Ref. Solo	47	59.6	1		
Duo	39	79.5	2.63 [0.99–6.95]		
Group (>2 PCPs)	81	71.6	1.71 [0.80–3.65]		
Participation in on-call duty – Ref No	87	66.7	1		
Yes	90	71.1	1.23 [0.65–2.33]		
Number of half-days worked per week^a^	177		1.05 [0.91–1.21]		
Seniority (in years)^a^	177		0.97 [0.93–0.99]		

a Included as a continuous variable, perceived health included 5 levels, from excellent = 1 to very bad = 5.

b % forgoing care due to heavy workload.

c Hosmer Lemeshow test, *p* = 0.83.

**TABLE 4:** Personal and professional factors associated with forgoing sick leave (N = 503, logistic regression) – [the Swiss Physicians’ Health Study, French-speaking Switzerland 2021].

Characteristics	N	%^b^	Univariate analyses	Multivariate analyses Final model^c^ (N = 484)	
			OR [95% CI]	OR [95% CI]	*p*-value
Sex – Ref. Male	242	24.8	1	1	
Female	257	33.1	1.50 [1.01–2.52]	1.59 [0.97–2.63]	0.07
Age – Ref. <45 years old	139	34.5	1	1	
45–54	146	27.4	0.72 [0.43–1.18]	0.64 [0.35–1.14]	0.13
55–64	141	32.6	0.92 [0.56–1.51]	1.07 [0.58–1.97]	0.82
≥65 years old	74	14.9	0.33 [0.16–0.69]	0.50 [0.21–1.19]	0.12
Marital status – Ref. Married	69	30.4	1		
Single	350	27.1	1.17 [0.67–2.06]		
Divorced	73	32.9	1.31 [0.76–2.26]		
Nationality – Ref. Swiss	315	24.8	1	1	
Naturalised Swiss citizen	117	36.7	1.77 [1.12–2.78]	1.99 [1.17–3.37]	0.01
Other	68	35.3	1.66 [0.95–2.90]	1.53 [0.80–2.92]	0.19
Perceived health – (Excellent to poor)^a^	498		3.09 [2.31–4.13]	2.50 [1.81–3.40]	0.01
Existing chronic disease – Ref No	253	19.8	1	1	
Yes, physical illness	144	23.8	1.27 [0.77–2.07]	1.33 [0.75–2.34]	0.33
Yes, psychological illness	104	58.6	5.76 [3.50–9.48]	3.79 [2.16–6.67]	0.01
Have their own doctor– Ref No	263	25.2	1	—	
Yes	237	33.3	1.48 [1.01–2.19]		
Setting – Ref. Urban	208	27.4	1		
Suburban area	190	27.9	1.02 [0.66–1.59]		
Rural area	102	34.3	1.38 [0.83–2.30]		
Speciality – Ref. General internal medicine	337	30.6	1		
Paediatrics	94	25.5	0.78 [0.46–1.31]		
Gynaecology	66	27.3	0.85 [0.47–1.54]		
Practice type – Ref. Solo	165	27.3	1		
Duo	105	25.7	0.92 [0.53–1.61]		
Group (>2 PCPs)	200	32.5	1.28 [0.82–2.01]		
Participation in on-call duty –Ref No	251	27.0	1		
Yes	248	31.0	1.21 [0.82–1.78]		
Number of half-days worked per week^a^	499		1.10 [1.06–1.20]	1.13 [1.01–1.26]	0.04
Seniority (in years)^a^	480		0.98 [0.94–1.00]		

a Included as a continuous variable, perceived health included 5 levels, from excellent = 1 to very bad = 5.

b % forgoing sick leave.

c Hosmer Lemeshow test, *p* = 0.46.

## Discussion

One third of the PCPs participating in our study had forgone care in the last year—a very significant proportion. The main reason reported for this illness behaviour was a high workload. Furthermore, about one third also adopted the illness behaviour of presenteeism, mainly because they did not want to abandon their patients, but financial reasons were also mentioned. Forgoing care because of a heavy workload (as opposed to all other causes) and forgoing sick leave were more common among those with certain demographic characteristics, particularly being a woman or a young PCP. Having one’s own GP was also associated with a higher frequency of forgoing care because of a heavy workload (as opposed to all other causes).

Our results were consistent with previous studies in terms of the scale of these problematic health behaviours among PCPs [13, 14]. Kay made a literature review of the barriers to seeking healthcare and categorised them into the “patient category” (including embarrassment, fear and time), the “provider category” (including confidentiality and quality of care) and the “system category” (including cultural and structural features) [13]. Our study revealed that a heavy workload was the commonest reason for forgoing care. A heavy workload and a lack of time also frequently appear in the literature but perhaps less often than the discomfort of being a patient [13]. However, our respondents had to choose from a predefined list of three reasons, which partly conditioned their response. They were free to propose another reason, if they wished, but only 9% did so (*n* = 15). Among the other reasons mentioned, only two GPs reported feelings of embarrassment. Finally, respondents making up excuses cannot be excluded. It may be easier or more self-gratifying for PCPs to report excess work than to acknowledge their fears or discomfort, even though the survey ensured their anonymity.

We observed no association between forgoing care and having one’s own GP. However, the heavy workload reason for forgoing care (vs. all other reasons) was linked to having a GP. PCPs without a GP responded more often with either “I did not need a medical consultation in the end” or “I treated the problem by myself” as their justification for forgoing care. This result suggests that these PCPs may be in denial of their illness or situation. It also reinforces international recommendations, particularly those of the British Medical Association [26, 27], about the importance of PCPs having their own GP. Less than half of the PCPs in our study had their own GP (47%). Montgomery’s literature review and meta-analysis, which confirmed the previous review by Kay [13], found a mean rate of PCPs with their own GP of 56%, ranging from 21 to 96%, depending on the study [14]; the lowest rate was in Switzerland [15]. In Schulz’s more recent study in Germany, 19% of GPs were registered as patients [20]. The characteristics of national healthcare systems, such as the existence of gatekeeping systems where patient registration with a GP is compulsory, can obviously affect this rate. However, because PCPs’ consultations are often informal, “corridor” consultations [13, 28], and because GPs often register with relatives [13], the exact effects of healthcare system characteristics remain unclear. In other words, mandatory registration with a GP will never be a complete solution to the problem of physicians’ illness behaviour.

Regarding forgoing sick leave, some research has been conducted on the specific domain of seeking treatment for psychological distress [17, 18, 29, 30]. In this context, the embarrassment or discomfort of being a psychologist’s patient or the fear of the opinions of colleagues and patients were particularly highlighted. Our results also point in this direction, showing that forgoing sick leave is more frequent in cases of psychological illness. The primary reason reported in our study was that PCPs did not want to abandon their patients. This is characteristic of PCPs’ professional dedication. However, financial reasons were also reported in half of the cases. This could also explain the associations observed with PCPs’ age. In a private primary care system like Switzerland’s, young PCPs may be more worried about financial aspects than their more experienced colleagues. However, the evolution of primary care’s organisation in many Western countries, including Switzerland, is towards more group practices and salaried GPs, and this might contribute to reducing the prevalence of this argument in the future [31].

Most of the factors associated with PCPs’ illness behaviours were sociodemographic characteristics and personal health attributes. Little association with professional characteristics was observed. However, items on those characteristics were limited in the questionnaire. It should be noted, nevertheless, that the number of half-days worked per week was positively associated with the frequency of forgoing sick leave. A kind of downward spiral of illness behaviour could result from this, with the doctors who work the most being those most at risk of exhaustion and also those who most neglect their health.

The results regarding age were not surprising—perhaps even reassuring—since older GPs are theoretically more vulnerable to illness. However, the results regarding sex were alarming. It should be noted that this result does not necessarily imply an absence of health follow-up among women, nor does it mean that women are more likely to forgo care than men (the difference was non-significant). It does mean that overwork was more frequently given as the reason why women forwent care. However, presenteeism was clearly associated with female sex, as Gustafsson Senden’s study also pointed out [32]. Davidson also reported sex differences in opinions and attitudes regarding illness behaviour: women physicians were more likely than men to report that finding a doctor was difficult [12]. The medical profession’s increasing feminisation and the often greater overall burden on women (in their private and professional lives) [33] raises fears that the negative impacts of these illness behaviours will increase over time.

Some authors have suggested the creation of a specific branch of healthcare delivery for physicians [19]. This might be an interesting response to illness behaviours surrounding confidentiality or embarrassment. However, it would probably not be sufficient. The valued and respected position of doctors in most societies probably helps to maintain the feelings of invincibility some of them experience (Kay’s “cultural barriers” [13]). The ongoing changes in patients’ attitudes towards the medical profession and doctors’ positions in society could also influence how physicians see themselves. In addition, work’s place and image in society are also changing, towards different ways of life and a better work–life balance. This phenomenon is also affecting highly dedicated professionals such as physicians [34]. Finally, the risks of engaging in these illness behaviours should probably be addressed during early medical training and then throughout careers, and on this last point, peer involvement and understanding would surely be valuable. According to Balme, “Doctors need to be supported, not trained in resilience” [35].

### Strengths and Limitations

Despite a good participation rate and good representativity in terms of age and medical specialities, we cannot exclude a selection bias. We were able to compare non-respondents with respondents in terms of sex and age and no differences were found. However, it was not possible to estimate a possible selection biais according to PCPs’ health status and interests in their own health. This could have led to an underestimation of the reported frequencies. In addition, our survey was done end of 2020, a year marked by the COVID-19 epidemic and particular strain on the health system. This might have impacted the prevalence rates of presenteeism and forgoing care. Still, we consider that this does not impact our conclusions. The reasons for forgoing care and sick leave were set, limiting participants’ possible responses. A qualitative approach would have been more appropriate to explore this issue further. In addition, providing a more in-depth analysis of how professional factors influence illness behaviours would have required information on more variables. However, this was not the survey’s specific topic. Another potential limitation is in the number of statistical tests which have been carried out and which may have led to too narrow *p*-values. However, when comparing the single-variable models to the selected multi-variable models, the main findings are quite similar which tends to show the robustness of our findings. Finally, because the study was cross-sectional, no causal conclusions can be drawn. One of the study’s strengths was that it focussed on PCPs, which, to the best of our knowledge, has never been done. Despite the fact that the study was conducted in Switzerland, we believe the results are probably generalisable to a large number of countries with comparable primary care systems, particularly European countries like France or Germany. Although there is some literature on barriers to healthcare access and doctors’ presenteeism [13], studies on the personal health determinants of such behaviours are scarce. Our study showed that female and young PCPs, in addition to those with chronic diseases, may be particularly prone to adopting harmful illness behaviours, especially presenteeism. Those working full-time were also more at risk. These results could be significant in helping to improve the effectiveness of preventive health interventions targeting this population.

### Conclusion

While it is important to have doctors who are highly dedicated to their work, healthcare systems must also ensure their sustainability and provide good quality care to patients. As part of this aim, the health and illness behaviours of primary care physicians are key issues. The evolution of primary care’s organisation combined with physicians’ gradually changing expectations of their work–life balance may have a positive impact on their future illness behaviours. Nevertheless, new generations of doctors—particularly young, female PCPs—seem reluctant to accord enough importance to their own health. They deserve careful monitoring, and specific solutions should be sought to reduce their tendencies to forgo care and sick leave.

## Key Points

• 37 and 29% of PCPs in French-speaking Switzerland reported at least one episode of forgoing care or sick leave, respectively;
• Presenteeism was more associated with female sex, younger age, having a chronic psychological illness, working in a suburb and working full-time;
• No associations between personal health characteristics and forgoing care were observed, but differences did exist between the reasons given for forgoing care.
