# Supplementary material for: Primary Care Physicians’ Personal and Professional Attributes Associated With Forgoing Own Care and Presenteeism: A Cross Sectional Study
Source: Int J Public Health. 2022 Feb 15;66:1604442. doi: 10.3389/ijph.2021.1604442 (PMC8886613; doi:10.3389/ijph.2021.1604442)
Supplement: Supplementary file 1 [file DataSheet1.pdf]

Appendix. List of medical conditions from the past five years and current medication evaluated in the study

**Past five years medical conditions**
Hypertension
Diabetes
Dyslipidemia
Obesity
Ischemic heart disease
Heart failure
Stroke or transient ischemic attack (TIA)
Peripheral arterial disease
Asthma
Chronic obstructive pulmonary disease (COPD)
Colon or rectal cancer
Prostate cancer
Breast cancer
Lung cancer
Other cancer (specify)
Depression and/or anxiety
Bipolar disorder
Burnout
**Current medication**
Pain
Paracetamol
Nonsteroidal anti-inflammatory drug (NSAID) or COX-2 inhibitor
Weak opioid
Gabapentin or pregabalin

At least one medication used
Cardiovascular disease
ACE inhibitor (ACEI) or angiotensin II receptor blocker (ARB)
Statin
Beta-blocker
Platelet anti-aggregant
Calcium antagonist
Diuretic
Anticoagulant
At least one medication used
Endocrine or bone disease
Calcium and vitamin D
Treatment of hypothyroidism
Metformin
At least one medication used
Psychiatric disease
Antidepressant
Benzodiazepine
Benzodiazepine analog
At least one medication used
Respiratory or allergic disease
Antihistamine
Inhaled corticosteroid
LABA, LAMA or SAMA
SABA
At least one medication used
Other
Proton-pump inhibitor (PPI)

Treatment of benign prostatic hyperplasia
Treatment of hyperuricemia
At least one medication used
Pain
Paracetamol
Nonsteroidal anti-inflammatory drug (NSAID) or COX-2 inhibitor
Weak opioid
Gabapentin or pregabalin
At least one medication used
Cardiovascular disease
ACE inhibitor (ACEI) or angiotensin II receptor blocker (ARB)
Statin
Beta-blocker
Platelet anti-aggregant
Calcium antagonist
Diuretic
Anticoagulant
At least one medication used
Endocrine or bone disease
Calcium and vitamin D
Treatment of hypothyroidism
Metformin
At least one medication used
Psychiatric disease
Antidepressant
Benzodiazepine
Benzodiazepine analog
At least one medication used

Respiratory or allergic disease
Antihistamine
Inhaled corticosteroid
LABA, LAMA or SAMA
SABA
At least one medication used
Other
Proton-pump inhibitor (PPI)
Treatment of benign prostatic hyperplasia
Treatment of hyperuricemia
At least one medication used
